# Response to “Being prepared to evaluate pregnancy PrEP”

**DOI:** 10.1002/jia2.25440

**Published:** 2019-12-24

**Authors:** Julia C Dettinger, John Kinuthia, Jillian Pintye, Jared M Baeten, Grace John‐Stewart

**Affiliations:** ^1^ Department of Global Health University of Washington Seattle WA USA; ^2^ Department of Obstetrics/Gynecology Kenyatta National Hospital Nairobi Kenya; ^3^ Department of Epidemiology University of Washington Seattle WA USA; ^4^ Department of Medicine University of Washington Seattle WA USA; ^5^ Department of Pediatrics University of Washington Seattle WA USA

**Keywords:** pre‐exposure prophylaxis, pregnancy, prevention, children, women, infant, Africa

We appreciate Dr. Slogrove's summary of the methodological limitations of programmatic data for assessing infant outcomes, and we concur with the need for a global consensus on acceptable strength of safety evidence for PrEP‐exposed pregnancies, as the safety threshold for a preventive agent is necessarily high. Following approval of PrEP for HIV prevention, the World Health Organization and countries both in Sub‐Saharan Africa and elsewhere developed guidelines that permit the use of PrEP in pregnancy by women at high risk for HIV acquisition 1, 2, 3, 4, 5, 6. Data to support that recommendation have come from the pivotal placebo‐controlled trials of PrEP, demonstration studies and registry information, and use of PrEP medications as part of HIV or HBV treatment 7; that body of evidence continues to increase, including with our PrIYA data 8. As for many medications, at the time of initial approval only limited safety data in pregnancy were available, with additional data often coming through small studies with wide confidence intervals.

We agree with Slogrove that we as a field should do better – intentionally considering pregnancy (and lactation) in the developmental pathway of new medications, gathering data on pregnancy safety sooner in the development of new HIV prevention (and treatment) modalities, and planning for large and rigorously conducted studies 9. As PrEP with TDF/FTC is recommended globally, thoughtful designs are necessary that preserve ethical access to PrEP, allowing women to choose whether or not to use that HIV prevention method. Some data gaps will be addressed in an ongoing cluster randomized trial evaluating PrEP delivery approaches in antenatal care (ANC) visits 10 (PrEP Implementation for Mothers in Antenatal Care (PrIMA, NCT03070600)). The PrIMA trial, which involves over 4000 mother‐infant pairs followed from pregnancy, will enable more rigorous assessment of infant outcomes by PrEP exposure, including stillbirth, miscarriage, birthweight, preterm birth, and infant growth through nine months. Further data will be obtained from a subset of PrIMA participants (approximately 1500) followed in an extension cohort that includes quantitative assessments of PrEP drug levels among both mothers and infants and follow‐up through five years of age, which includes bone mineralization, neurocognitive, and growth outcomes (R01HD100201).

Finally, in addition to these studies, as PrEP is delivered to women in settings with large numbers of exposed pregnancies, it is important to continue to compile data for rare infant outcomes that can only be adequately addressed through large, program databases 11.

## Competing interests

The authors have no conflicts of interest to declare.

## Authors' contributions

JCD, JP and GJS drafted the response. JK and JMB reviewed and provided revisions to the draft and approved of the final letter.

## Abbreviations

ANC, antenatal care; PrEP, pre‐exposure prophylaxis; PrIMA, PrEP Implementation for Mothers in Antenatal Care.
